# An extended reinforcement learning model of basal ganglia to understand the contributions of serotonin and dopamine in risk-based decision making, reward prediction, and punishment learning

**DOI:** 10.3389/fncom.2014.00047

**Published:** 2014-04-16

**Authors:** Pragathi P. Balasubramani, V. Srinivasa Chakravarthy, Balaraman Ravindran, Ahmed A. Moustafa

**Affiliations:** ^1^Department of Biotechnology, Indian Institute of Technology - MadrasChennai, India; ^2^Department of Computer Science and Engineering, Indian Institute of Technology - MadrasChennai, India; ^3^Foundational Processes of Behaviour Research Concentration, Marcs Institute for Brain and Behaviour & School of Social Sciences and Psychology, University of Western SydneySydney, NSW, Australia

**Keywords:** serotonin, dopamine, basal ganglia, Reinforcement Learning, Risk, Reward, Punishment, Decision Making

## Abstract

Although empirical and neural studies show that serotonin (5HT) plays many functional roles in the brain, prior computational models mostly focus on its role in behavioral inhibition. In this study, we present a model of risk based decision making in a modified Reinforcement Learning (RL)-framework. The model depicts the roles of dopamine (DA) and serotonin (5HT) in Basal Ganglia (BG). In this model, the DA signal is represented by the temporal difference error (δ), while the 5HT signal is represented by a parameter (α) that controls risk prediction error. This formulation that accommodates both 5HT and DA reconciles some of the diverse roles of 5HT particularly in connection with the BG system. We apply the model to different experimental paradigms used to study the role of 5HT: (1) Risk-sensitive decision making, where 5HT controls risk assessment, (2) Temporal reward prediction, where 5HT controls time-scale of reward prediction, and (3) Reward/Punishment sensitivity, in which the punishment prediction error depends on 5HT levels. Thus the proposed integrated RL model reconciles several existing theories of 5HT and DA in the BG.

## Introduction

Monoamine neuromodulators such as dopamine, serotonin, norepinephrine and acetylcholine are hailed to be the most promising neural messengers to ensure healthy adaptation to our uncertain environments. Specifically, serotonin (5HT) and dopamine (DA) play important roles in various cognitive processes, including reward and punishment learning (Cools et al., 2011; Rogers, 2011). DA signaling has been linked to reward processing in the brain for a long time (Bertler and Rosengren, 1966). Furthermore the activity of mesencephalic DA neurons are found to closely resemble temporal difference error (TD) in Reinforcement Learning (RL) (Schultz, 1998). This TD error represents the difference in the total reward (outcome) that the agent or subject receives at a given state and time, and the total predicted reward. The semblance between the TD error signal and DA signal served as a starting point of an extensive theoretical and experimental effort to apply concepts of RL to understand the functions of the Basal Ganglia (BG) (Schultz et al., 1997; Sutton and Barto, 1998; Joel et al., 2002; Chakravarthy et al., 2010). This led to the emergence of a framework for understanding the BG functions in which the DA signal played a crucial role. Deficiency of such a neuromodulator (DA) leads to symptoms observed in neurodegenerative disorders like Parkinson's Disease (Bertler and Rosengren, 1966; Goetz et al., 2001).

### The multiple functions of serotonin

It is well-known that dopamine is not the only neuromodulator that is associated with the BG function. Serotonin (5HT) projections to the BG are also known to have an important role in decision making (Rogers, 2011). 5HT is an ancient molecule that existed even in plants (Angiolillo and Vanderkooi, 1996). Through its precursor tryptophan, 5HT is linked to some of the fundamental processes of life itself. Tryptophan-based molecules in plants are crucial for capturing the light energy necessary for glucose metabolism and oxygen production (Angiolillo and Vanderkooi, 1996). Thus, by virtue of its fundamental role in energy conversion, 5HT is integral to mitosis, maturation, and apoptosis. In lower organisms, it modulates the feeding behavior and other social behaviors such as dominance posture, and escape responses (Kravitz, 2000; Azmitia, 2001; Chao et al., 2004). Due to its extended role as a homeostatic regulator in higher animals and in mammals, 5HT is also associated with appetite suppression (Azmitia, 1999; Halford et al., 2005; Gillette, 2006). Furthermore, 5HT plays important roles in anxiety, depression, inhibition, hallucination, attention, fatigue, and mood (Tops et al., 2009; Cools et al., 2011). Increasing 5HT level leads to decreasing punishment prediction, though recent evidence pointing to the role of DA in processing aversive stimuli makes the picture more complicated (So et al., 2009; Boureau and Dayan, 2011). The tendency to pay more attention to negative than positive experiences or other kinds of information (negative cognitive biases) are found to occur at lower levels of 5HT (Cools et al., 2008; Robinson et al., 2012). 5HT is also known to control the time scale of reward prediction (Tanaka et al., 2007) and to play a role in risk sensitive behavior (Long et al., 2009; Murphy et al., 2009; Rogers, 2011). Studies found that under conditions of tryptophan depletion, which is known to reduce the brain 5HT level, risky choices are preferred to safer ones in decision making tasks (Long et al., 2009; Murphy et al., 2009; Rogers, 2011). Reports about 5HT transporter gene influencing risk based decision making also exist (He et al., 2010; Kuhnen et al., 2013). 5HT is known to influence non-linearity in risk-based decision making (Kahneman and Tversky, 1979)—risk-aversivity in the case of gains and risk-seeking during losses, while presented with choices of equal means (Murphy et al., 2009; Zhong et al., 2009a,b). In summary, 5HT is not only important for behavioral inhibition, but is also related to time scales of reward prediction, risk, anxiety, attention etc., and to non-cognitive functions like energy conversion, apoptosis, feeding, and fatigue.

### Prior theoretical and computational abstract models of serotonin

It would be interesting to understand and reconcile the roles of DA and 5HT in the BG. Prior abstract models addressing the same quest such as that by Daw et al. (2002) argue that DA signaling plays a role that is complementary to 5HT. It has been suggested that whereas the DA signal responds to appetitive stimuli, 5HT responds to aversive or punitive stimuli (Daw et al., 2002). Unlike computational models that argue for complementary roles of DA and 5HT, empirical studies show that both neuromodulators play cardinal roles in coding the signals associated with the reward (Tops et al., 2009; Cools et al., 2011; Rogers, 2011). Genes that control neurotransmission of both molecules are known to affect processing of both rewarding and aversive stimuli (Cools et al., 2011). Complex interactions between DA and 5HT make it difficult to tease apart precisely the relative roles of the two molecules in reward evaluation. Some subtypes of 5HT receptors facilitate DA release from the midbrain DA releasing sites, while others inhibit them (Alex and Pehek, 2007). In summary, it is clear that the relationship between DA and 5HT is not one of simple complementarity. Both synergistic and opposing interactions exist between these two molecules in the brain (Boureau and Dayan, 2011).

Efforts have been made to elucidate the function of 5HT through abstract modeling. Daw et al. (2002) developed a line of modeling that explores an opponent relationship (Daw et al., 2002; Dayan and Huys, 2008) between DA and 5HT. In an attempt to embed all the four key neuromodulators—DA, 5HT, norepinephrine and acetylcholine—within the framework of RL, Doya (2002) associated 5HT with discount factor, γ, which is a measure of time-scale of reward integration (Doya, 2002; Tanaka et al., 2007). There is no single computational theory that integrates and reconciles the existing computational perspectives of 5HT function in a single framework.

### Our model in brief

In this modeling study, we present a model of both 5HT and DA in BG simulated using a modified RL framework. Here, DA represents TD error as in most extant literature of DA signaling and RL (Schultz et al., 1997; Sutton and Barto, 1998), and 5HT controls risk prediction error. Action selection is controlled by the utility function that is a weighted combination of both the value and risk function (Bell, 1995; Preuschoff et al., 2006; D'acremont et al., 2009). In the proposed modified formulation of utility function, the weight of the risk function depends on the sign of the value function and a tradeoff parameter α, which we describe in detail below. Just as value function was thought to be computed in the striatum, we now propose that the utility function is computed in the striatum.

The outline of the paper is as follows: Section Methods describes the model equations. In Section Results, we show that a combination of both value and the risk function for decision making explains the following experiments. The first of these pertains to risk sensitivity in bee foraging (Real, 1981). Here we demonstrate that the proposed 5HT and DA model can simulate this simple neurobiological instance of risk-based decision making. We then show the capability of the model to explain the roles of 5HT in the representative experimental conditions: risk sensitivity in Tryptophan depleted conditions (Long et al., 2009); time-scale of reward prediction (Tanaka et al., 2007); and reward and punishment sensitivity (Cools et al., 2008). We present the discussion on the model and results in Section Discussion. Furthermore in the discussion, we hypothesize that the plausible neural correlates for the risk component are the D1R and the D2R co-expressing medium spiny neurons of the striatum, with serotonin selectively modulating this population of neurons.

## Methods

On the lines of the utility models described by Bell (1995) and D'acremont et al. (2009), we present here the utility function, *U_t_* as a tradeoff between the expected payoff and the variance of the payoff (the subscript *“t”* refers to time). The original Utility formulation used in Bell (1995; D'acremont et al. (2009) is (Equation 2.1).

(2.1) $$U_{t}(s,\;a)=Q_{t}(s,\;a)-\kappa \sqrt{h_{t}(s,\;a)}$$

where *Q_t_* is the expected cumulative reward and *h_t_* is the risk function or reward variance, for state, *s*, action, *a*; κ is the risk preference. Note that in equation. 2.1, we represent the state and action explicitly as opposed to (Bell, 1995; D'acremont et al., 2009).

In classical RL (Sutton and Barto, 1998) terms, following policy, π, the action value function, *Q*, at time *t* of a state, *“s,”* and action, *“a”* may be expressed as (Equation 2.2).

(2.2) $$Q^{\pi }(s,\;a)=E_{\pi }(r_{t\;+\;1}+\gamma r_{t\;+\;2}+\gamma^{2}r_{t\;+\;3}+\cdots |s_{t}=s,\;a_{t}=a)$$

where *r_t_* is the reward obtained at time, *t*, and γ is the discount factor (0 < γ < 1). *E*_π_ denotes the expectation when action selection is done with policy π. The incremental update for the action value function, *Q* is defined as in Equation 2.3.

(2.3) $$Q_{t\;+\;1}(s_{t},\;a_{t})=Q_{t}(s_{t},\;a_{t})+\eta_{Q}\delta_{t}$$

where *s_t_* is the state at time, *t*; *a*_t_ is the action performed at time, *t*, and η_*Q*_ is the learning rate of the action value function (0 < η_*Q*_ < 1). δ_*t*_ is the TD error defined by Equation 2.4,

(2.4) $$\delta_{t}=r_{t\;+\;1}+\gamma Q_{t}\left(s_{t\;+\;1},\;a_{t+1}\right)-Q_{t}\left(s_{t},\;a_{t}\right)$$

In the case of immediate reward problems, δ_*t*_ is defined by Equation 2.5.

(2.5) $$\delta_{t}=r_{t}-Q_{t}\left(s_{t},\;a_{t}\right)$$

Similar to the value function, the risk function “*h_t_*” has an incremental update as defined by Equation 2.6.

(2.6) $$h_{t\;+\;1}(s_{t},\;a_{t})=h_{t}(s_{t},\;a_{t})+\eta_{h}\xi_{t}$$

where η_*h*_ is the learning rate of the risk function (0 < η_*h*_ < 1), and ξ_*t*_ is the risk prediction error expressed by Equation 2.7,

(2.7) $$\xi_{t}=\delta_{t}^{2}-h_{t}(s_{t},\;a_{t})$$

η_*h*_ and η_*Q*_ are set to 0.1, and *Q_t_* and *h_t_* are set to zero at *t* = 0 for simulations of (sections Risk Sensitivity and Rapid Tryptophan Depletion, Time Scale of Reward Prediction and Serotonin, Reward/Punishment Prediction Learning and Serotonin) described below.

We now present a modified form of the utility function by substituting κ = α.*sign*[*Q_t_*(*s_t_*, *a_t_*)] in (Equation 2.1).

(2.8) $$U_{t}(s_{t},\;a_{t})=Q_{t}(s_{t},\;a_{t})-\alpha sign(Q_{t}(s_{t},\;a_{t}))\sqrt{h_{t}(s_{t},\;a_{t})}$$

In (Equation 2.8), the risk preference includes three components—the “α” term, the “*sign(Q_t_)”* term, and the risk term $\sqrt{h_{t}}$. The *sign(Q_t_)* term achieves a familiar feature of human decision making viz., risk-aversion for gains and risk-seeking for losses (Kahneman and Tversky, 1979). In other words, when *sign(Q_t_)* is positive (negative), *U_t_* is maximized (minimized) by minimizing (maximizing) risk. Note that the expected action value *Q_t_* would be positive for gains that earn rewards greater than a reward base (= 0), and would be negative otherwise during losses. We associate 5HT level with α, a constant that controls the relative weightage between action value and risk (Equation 2.8).

In this study, action selection is performed using softmax distribution (Sutton and Barto, 1998) generated from the utility. Note that traditionally the distribution generated from the action value is used. The probability, *P_t_(a|s)* of selecting an action, *a*, for a state, *s*, at time, *t*, is given by the softmax policy (Equation 2.9).

(2.9) $$P_{t}(a|s)=exp(\beta U_{t}(s,\;a))/\sum_{i\;=\;1}^{n}\exp(\beta U_{t}(s,i))$$

*n* is the total number of actions available at state, *s*, and β is the inverse temperature parameter. Values of β tending to 0 make the actions almost equiprobable and the β tending to ∞ make the softmax action selection identical to greedy action selection.

## Results

In this section, we apply the model of 5HT and DA in BG (Section Methods) to explain several risk-based decision making phenomena pertaining to BG function.

1. Measurement of risk sensitivity: Two experiments are simulated in this category:
  - Risk sensitivity in Bee foraging (Real, 1981)
  - Risk sensitivity and Tryptophan depletion (Long et al., 2009)
2. Representation of time scale of reward prediction (Tanaka et al., 2007) and
3. Measurement of punishment sensitivity (Cools et al., 2008).

The parameters for each experiment are optimized using genetic algorithm (GA) (Goldberg, 1989) (Details of the GA option set are given in Supplementary material).

### Risk sensitivity in bee foraging

#### Experiment summary

In the bee foraging experiment by Real (1981), bees were allowed to choose between flowers of two colors—blue and yellow. Both types of flowers deliver the same amounts of mean reward (nectar) but differ in the reward variance. The experiment showed that bees prefer the less risky flowers i.e., the one with lesser variance in nectar (Real, 1981).

Biogenic amines such as 5HT are found to influence foraging behavior in bees (Schulz and Robinson, 1999; Wagener-Hulme et al., 1999). In particular, the brain levels of dopamine, serotonin, and octopamine are found to be high in foraging bees (Wagener-Hulme et al., 1999). Montague et al. (1995) showed risk aversion in bee foraging using a general predictive learning framework without mentioning DA. They assume a special “subjective utility” which is a non-linear reward function (Montague et al., 1995) to account for the risk sensitivity of the subject. In the foraging problem of (Real, 1981) bees choose between two flowers that have the same mean reward but differ in risk or reward variance. Therefore, the problem is ideally suited for risk-based decision making approach. We show that the task can be modeled, without any assumptions about “subjective utility,” by using the proposed 5HT-DA model which has an explicit representation for risk.

#### Simulation

We model the above phenomenon of bee foraging using the modified utility function of Section Methods. This foraging problem of (Real, 1981) is treated as a variation of the stochastic “two-armed bandit” problem (Sutton and Barto, 1998), possessing no state (*s*) and 2 actions (*a*). We represent the colors of the flower (“yellow” and “blue”) that happens to be the only predictor of nectar delivery as two arms (viz. the two actions, *a*). Initial series of experimental trials is modeled to have all the blue flowers (“no-risk” choice) delivering 1 μl (reward value, *r* = 1) of nectar; 1/3 of the yellow flowers delivering 3 μl (*r* = 3), and the remaining 2/3 of the yellow flowers contain no nectar at all (*r* = 0) (yellow flowers = “risky” choice). These contingencies are reversed at trial 15 and stay that way till trial 40. Since the task here requires only a single decision per trial, we model it as an *immediate reward* problem (Equation 2.5). Hence the δ for any trial *t* is calculated as in Equation 3.1.2.1 for updating the respective action value by Equation 3.1.2.2.

(3.1.2.1) $$\delta_{t}\;=r_{t}-Q_{t}\left(a_{t}\in \{blueflower,yellowflower\}\right)$$

(3.1.2.2) $$Q_{t+1}(a_{t})=Q_{t}(a_{t})+\eta_{Q}\delta_{t}$$

(3.1.2.3) $$h_{t+1}(a_{t})=h_{t}(a_{t})+\eta_{h}\xi_{t}$$

(3.1.2.4) $$\xi_{t}\;=\delta_{t}^{2}-h_{t}(a_{t})$$

(3.1.2.5) $$U_{t}(a_{t})=Q_{t}(a_{t})-\alpha sign(Q_{t}(a_{t}))\sqrt{h_{t}(a_{t})}$$

In our simulation, the expected action value (given by *Q*) for both the flowers converges to be the same value (=1). Our model accounts for the risk through the variance (represented by “*h*” of each flower: Equations 3.1.2.3, 3.1.2.4) component in the utility function (Equation 3.1.2.5) that plays a key role in the action selection.

#### Results

In the experiment (Real, 1981), most of the bees visited the constant nectar yielding blue flowers initially i.e. they chose a risk-free strategy, but later the choice switched to the yellow flowers, once the yellow became the less risky choice. We observe the same in our simulations too. Risk-aversive behavior being an optimal approach during the positive rewarding scenario, the blue flowers that deliver a steady reward of 1 have higher utility and are preferred over the more variable yellow flowers initially. The situation is reversed after trial 15 when the blue flowers suddenly become risky and the yellow ones become risk-free. Here, the utility of the yellow flowers starts increasing, as expected. Note that the expected action value for both flowers still remains the same, though the utility has changed.

**Figure 1:**
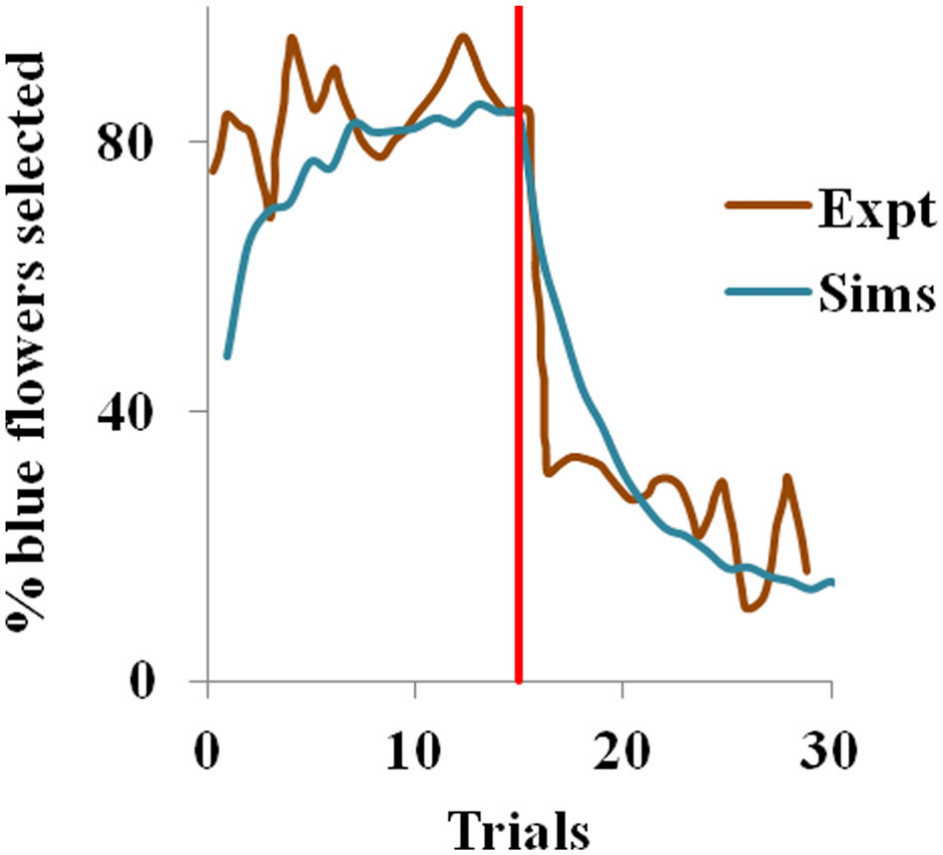
**Selection of the blue flowers obtained from our simulation (Sims) as an average of 1000 instances, that adapted from Real (1981) experiment (Expt), and red line indicating contingency reversal**.

With η_*h*_ = 0.051, η_*Q*_= 0.001, α = 1.5 in Equation 3.1.2.5, and β = 10 in Equation 2.9 for the simulation, the proposed model captures the shift in selection in less than 5 trials from the indication of the contingency reversal (red line in the Figure 1). Since the value is always non-negative, and α > 0, our model exhibits risk-averse behavior, similar to the bees in the study.

### Risk sensitivity and rapid tryptophan depletion

#### Experiment summary

Now we show that the above risk based decision making by 5HT-DA model framework can also explain the Long et al. (2009) experiment on risk sensitivity under conditions of Tryptophan depletion. Their experiment required the monkey to saccade to one of two given targets. One target was associated with a guaranteed juice reward (safe) and the other with a variable juice volume (risky). A non-linear risk sensitivity toward juice rewards by adopting risk-seeking behavior for small juice rewards and risk aversive behavior for the larger ones (Long et al., 2009) was observed in the monkeys. They showed that when brain 5HT levels are reduced by Rapid Tryptophan Depletion (RTD), monkeys preferred risky over safer alternatives (Long et al., 2009). Tryptophan acts as a precursor to 5HT and therefore reduction in tryptophan causes reduction in 5HT.

#### Simulation

The juice rewards *r^j^*, represented in Long et al. (2009) as open time of the solenoid used to control the juice flow to the mouth of the monkeys, are given in Table 1. The non-linearity in risk attitudes observed by the monkeys is accounted for in the model by considering a reward base (*r^b^*) that is subtracted from the juice reward (*r^j^*) obtained. The resultant subjective reward *(r)* is treated as the actual immediate reward received by the agent (Equation 3.2.2.1). Subtracting *r^b^* from *r^j^*, associates any *r^j^* < *r^b^* with an effect similar to losses (economy), and any *r^j^* > *r^b^* with gains.

(3.2.2.1) $$r=r^{j}-r^{\;b}$$

**Table 1:** **The sample reward schedule adapted from Long et al. (2009)**.

**Serial no.**	**Safe target (ms)**	**Risky targets (ms)—each with probability 0.5**
**(STATES, “*s*”)**	**(*r*^j^)**	
1	150	125,175
2	150	100,200
3	150	50,250
4	140	40,240
5	200	40,240
6	210	40,240

The reward base (*r^b^*) used in the experiment is 193.2. A separate utility function *U_t_*, is computed using Equation 2.8 for each state *'s'* tabulated in (Table 1) and action choice, *a* (a∈{safe target, risky target}) pair. This is also modeled as an *immediate reward* problem and the subjective reward given by Equation 3.2.2.1 is used for the respective (state, action) pair's TD error calculation (Equation 2.5). The action value function is updated over trials using Equation 2.3 and the risk updates are using Equation 2.6 for any (state, action) pair described above.

#### Results

**Figure 2:**
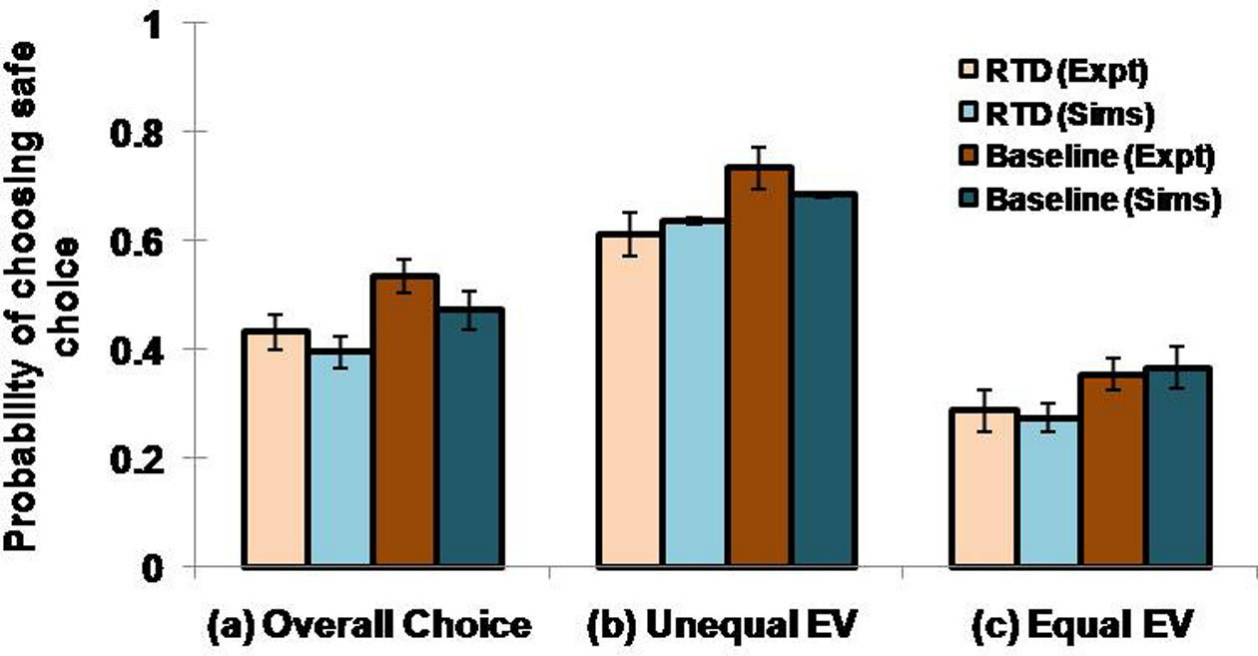
**Comparison between the experimental and simulated results for the (A) overall choice (B) Unequal EV (C) Equal EV, under RTD and Baseline (control) condition.** Error bars represent the SE with size “*N*” = 100.The experiment (Expt) and the simulation (Sims) result of any condition did not reject the null hypothesis, which proposes no difference between means, with *P* value > 0.05. Here the experimental results are adapted from Long et al. (2009).

Here we examine the following conditions: (1) overall choice, (2) equal expected value (EEV) and (3) unequal expected value (UEV). In EEV cases, saccade to either the safe or the risky target offered the same mean reward, as shown in the first four states (*s*) of the (Table 1). In UEV cases, the mean reward maintained for the two targets is not the same, as in the last two states (*s*) of the (Table 1). The optimized 5HT parameter (used in Equation 2.8), α, is equal to 1.658 for the RTD condition and is 1.985 for the baseline (control) condition. The optimized β used in Equation 2.9 is 0.044. Long et al. (2009) demonstrated a significant reduction in choosing safe option on lowering the 5HT levels in brain. This was seen irrespective of the options possessing equal or unequal expected value (EEV/ UEV). Our simulation results also generated a similar trend for EEV and UEV conditions (Figure 2: Sims) as that of experimental results [Figure 2: expt adapted from Long et al. (2009)]. The classical RL model would fail to account for such a result in the selection of safe option especially in the EEV case, where that model would predict equal probability (= 0.5) for selecting both the safe and risky rewards.

### Time scale of reward prediction and serotonin

#### Experiment summary

In this section, we show using the model of Section Methods that the α parameter that represents 5HT is analogous to the time-scale of reward integration (γ as in Equation 2.2) as described in the experiment of Tanaka et al. (2007). In order to verify the hypothesis that 5HT corresponds to the discount factor, γ (as in Equation 2.4), Tanaka et al. (2007) conducted an experiment in which subjects performed a multi-step delayed reward choice task under an fMRI scanner. Subjects had to choose between a white square leading to a small early reward and a yellow square leading to a large but delayed reward (Tanaka et al., 2007). They were tested in: (1) tryptophan depleted, (2) control and (3) excess tryptophan conditions. At the beginning of each trial, subjects were shown two panels, each consisting of white and yellow squares, respectively. The two panels were occluded by variable numbers of black patches. When the subjects selected any one of the panels, a variable number of black patches are removed from the selected panel. When either panel was completely exposed, reward was provided. One of the panels (yellow) provided larger reward with greater delay; the other (white) delivered a smaller reward but after a shorter delay. A total of 8 trials were presented to each subject and the relative time delay ranges set for the white and the yellow panels are (3.75~11.25 s, 15~30 s) in four trials, (3.75~11.25 s, 7.5~15 s) in two trials, and (1.6~4.8 s, 15~30 s) and (1.6~4.8 s, 7.5~15 s) in one trial each.

#### Simulation

We modeled the above task with the state variable, *s*, representing the number of black patches in a panel and action, *a*, as choosing any one of the panels. Each simulation time step equals one experimental time step of 2.5 s. The initial number of black patches on the white and yellow panels are 18 ± 9, and 72 ± 24 respectively. The number of patches removed varied between trials, and are given for the white panel and the yellow panel as follows (Tanaka et al., 2007). They are (S_s_, S_l_) = (6 ± 2, 8 ± 2) in 4 trials, (6 ± 2, 16 ± 2) in 2 trials, and (14 ± 2, 8 ± 2), (14 ± 2, 16 ± 2) in the remaining 2 trials respectively. The above 8 trials are repeated for all three tryptophan conditions viz. depleted, control and excess. Finally the reward associated with the white panel is *r* = 1 and with that of yellow is *r* = 4. Since there is a delay in receiving the reward, the TD error formulation used in Equation 3.3.2.1 is used for updating the value of the states (denoting the discounted expectation of reward from a particular number of patches in a panel). The action of removing certain patches from a panel actually leads to another resultant state with a reduced number of patches. Hence at any particular “*t*” the resultant states of white and yellow panels are compared for action selection. While the value function is updated using Equation 3.3.2.2, the risk function is updated as in Equations 3.3.2.3, 3.3.2.4. The agent is then made to choose between the utility functions given by Equation 3.3.2.5 of both the panels at time, *t*. Eventually the panel that is completely exposed is labeled as selected for a particular trial.

(3.3.2.1) $$\delta_{t}=r_{t\;+\;1}+\gamma Q_{t}\left(s_{t\;+\;1}\right)-Q_{t}\left(s_{t}\right)$$

(3.3.2.2) $$Q_{t\;+\;1}(s_{t})=Q_{t}(s_{t})+\eta_{Q}\delta_{t}$$

(3.3.2.3) $$h_{t\;+\;1}(s_{t})=h_{t}(s_{t})+\eta_{h}\xi_{t}$$

(3.3.2.4) $$\xi_{t}=\delta_{t}^{2}-h_{t}(s_{t})$$

(3.3.2.5) $$U_{t}(s_{t})=Q_{t}(s_{t})-\alpha sign(Q_{t}(s_{t}))\sqrt{h_{t}(s_{t})}$$

#### Results

**Figure 3:**
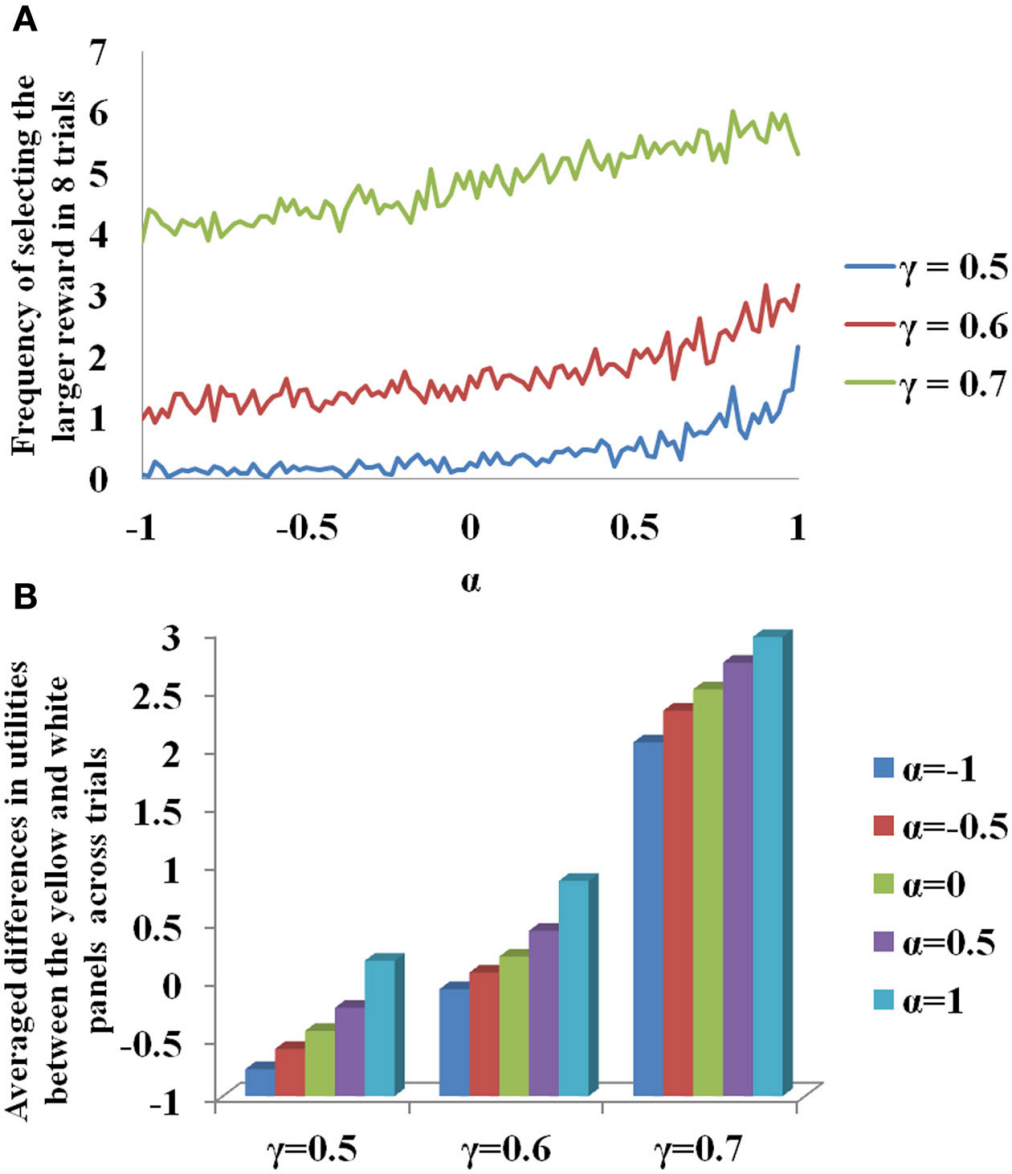
**(A)** Selection of the long term reward as a function of α. Increasing γ increased the frequency of selecting the larger and more delayed reward. Increasing α also gave similar results for a fixed γ. **(B)** Differences in the utilities (*U*) between the yellow and white panels averaged across trials for the states, *s_t_*, as a function of γ and α. Here *N* = 2000.

In Figure 3A, for sample values of γ = (0.5, 0.6, 0.7) used in Equation 3.3.2.1, the probability of selecting larger reward is plotted as a function of α. Note that for constant γ, the probability of selecting delayed reward increases with α. The β used to report the Figure 3 is 20. The change of value (*Q*) and risk function (*h*) as a function of the *states, s* (# of black patches) of each panel is shown in Supplementary material for various values of γ. If α is interpreted as 5HT level, delayed deterministic reward choices are favored at higher 5HT levels. Thus α in our model effectively captures the role of γ in the experiment of Tanaka et al. (2007) for functionally representing the action of 5HT in the striatum of BG. In addition, a trend of increasing differences between the utilities of the yellow and the white panels as a function of the state, *s_t_*, could be seen on increasing the value of α (Figure 3B). This is similar to the increasing differences of value functions for states, *s_t_*, between the yellow and white panels on increasing the value of γ (Figure 3B, Supplementary material). These differences in values / utilities are of prime importance for deciding the exploration/exploitation type of behavior by any policy such as that in Equation 2.9.

Our goal in the Section Time Scale of Reward Prediction and Serotonin is to relate our model's serotonin correlate (α in Equation 2.8) to that proposed in experiment of Tanaka et al. (2007) (γ as in Equation 2.2) in striatum. The differential activity of striatum observed in fMRI of the subjects in different tryptophan conditions was indeed modeled in Tanaka et al. (2007) via value functions (Equations 2.2–2.3) with different γ values. Specifically, the value generated by a lower (higher) γ value better modeled the striatal activity following tryptophan depletion (excess tryptophan). An increase in γ results in a value distribution, which when expressed with a particular value of β (Equation 2.9), would increase the probability of selecting the delayed but larger rewards (Sutton and Barto, 1998). Note that the subjects in Tanaka et al. (2007) show no great preference to one action over the other, though the striatal activity levels in subjects show sensitivity to γ values. This could be because action selection is not singularly influenced by the striatum and is probably influenced by downstream structures like GPi (Globus Pallidus—interna), or parallel structures like STN (SubThalamic Nucleus) and GPe (Globus Pallidus—externa) (Chakravarthy et al., 2010). Doya (2002) suggested that the randomness in action selection, which has been parametrized by β (Equation 2.9) in RL models, can be correlated by the effect of norepinephrine on the Pallidum. Thus for sufficiently small β, it is possible to obtain equal probability of action selection, though the corresponding utilities might sufficiently different. The focus of this section is to draw analogies between the discount parameter γ of classical RL models, and α parameter in our utility-based model, as substrates for *5HT function in striatum*.

### Reward/punishment prediction learning and serotonin

#### Experiment summary

The ability to differentially learn and update action selection by reward and punishment feedback is shown to change on altering the tryptophan levels in subjects. We model a deterministic reversal learning task (Cools et al., 2008; Robinson et al., 2012) in which the subjects were presented with two stimuli, one associated with reward and the other with punishment. On each trial, the subjects had to predict whether the highlighted stimulus would lead to reward or punishment response. The subjects were tested in either a balanced or a depleted tryptophan levels (drink), on their association of the stimulus to the corresponding action at any time. Erroneous trials were followed by the same stimulus till it has been predicted by the subject correctly and the same is adopted in the simulations too. Trials were grouped into blocks. Each subject performed 4 experimental blocks, which were preceded by a practice block in order to familiarize the subject with the task. Each experimental block consisted of an acquisition stage followed by a variable number of reversal stages. One of two possible experimental conditions was applied to each block. The experimental conditions were: unexpected reward (punishment) condition where a stimulus previously associated with punishment (reward) becomes rewarding (punishing). Since there are 4 blocks of trials, there were two blocks for each condition. Performance of the subjects in the non-reversal trials was evaluated as a function of—(a) drink and condition (unexpected reward/unexpected punishment), and (b) drink and outcome (reward/punishment) trial type. Results showed that performance did not vary significantly with condition in both balanced and tryptophan depleted cases. Errors were fewer for tryptophan depleted cases than balanced cases in both conditions. Specifically, errors were fewer for punishment-prediction trials compared to reward-prediction trials in tryptophan-depleted cases. Thus the experiment suggests that tryptophan-depletion selectively enhances punishment-prediction relative to reward-prediction. Please refer (Cools et al., 2008) for a detailed explanation of the experimental setup and results.

#### Simulation

We model the two stimuli as states, *s* ($s\in \left\{s_{1},\;s_{2}\right\}$), and the response of associating a stimulus to reward or punishment as action, *a* (action $a\in \left\{a_{1}=reward,\;a_{2}=punishment\right\}$). At any particular trial, *t*, the rewarding association is coded by *r_t_*= +1, and the punitive association is coded by *r_t_* = −1. This is treated as an immediate reward problem and the TD error calculation in Equation 2.5 is used. As in the experiments, three types of trials are simulated as follows: non-reversal trials in which the association of a stimulus—response pair is learnt; reversal trials in which the change of the learnt association is triggered; and the switch trials where the reversed associations are tested following the reversal trials. The setup followed is similar to that of the experiment: The maximum numbers of reversal stages per experimental block are 16, with each stage to continue till the correct responses fall in the range of (5–9). The block terminates automatically after 120. There are two blocks in each condition, and hence a total of 480 trials (4 blocks) conducted per agent. The design of the experiment has an inbuilt complementarity in the association of the actions to a particular stimulus (increasing the action value of *a*_1_ for a stimulus, *s*, decreases the same of *a*_2_ to *s*) and that of the stimuli to a particular action (increasing the action value of *s*_1_ to *a* decreases the same for *s*_2_ to *a*). Hence in the simulations, the action values associated [*Q_t_*(*s_t_*, *a_t_*) as in Equation 2.3] with the two actions [*Q*(*s*, *a_t_*) and *Q*(*s_t_*,*a_2_*)] for any particular state *'s'* are simulated to be complimentary (Equation 3.4.2.1) at any trial “*t.*”

(3.4.2.1) $$Q(s,\;a_{1})=-Q(s,\;a_{2})$$

The action values of the two stimuli, *s*, [*Q*(*s_1_*, *a*) and *Q*(*s_2_*,*a*)] mapped to the same action, *a* are also complimentary (Equation 3.4.2.2) at any trial, “*t.*”

(3.4.2.2) $$Q(s_{1},\;a)=-Q(s_{2},\;a)$$

Hence, only one out of the four value functions [*Q*(s_1_, a_1_), *Q*(s_1_, a_2_), *Q*(s_2_, a_1_), *Q*(s_2_, a_2_),] are learnt by training while the other 3 are set by the complementarity rules to capture the experimental design. We assume that such a complementarity could be learnt during the initial practice block that facilitated familiarity. The action (response) selection is by setting the β of the policy Equation 2.9 optimized to 10, and executing the same policy on the utilities (Equation 2.8) of the two responses (*a*) for any given stimulus (*s*) at a trial (*t*). The risk functions for the same are given by Equation 2.6.

#### Results

**Figure 4:**
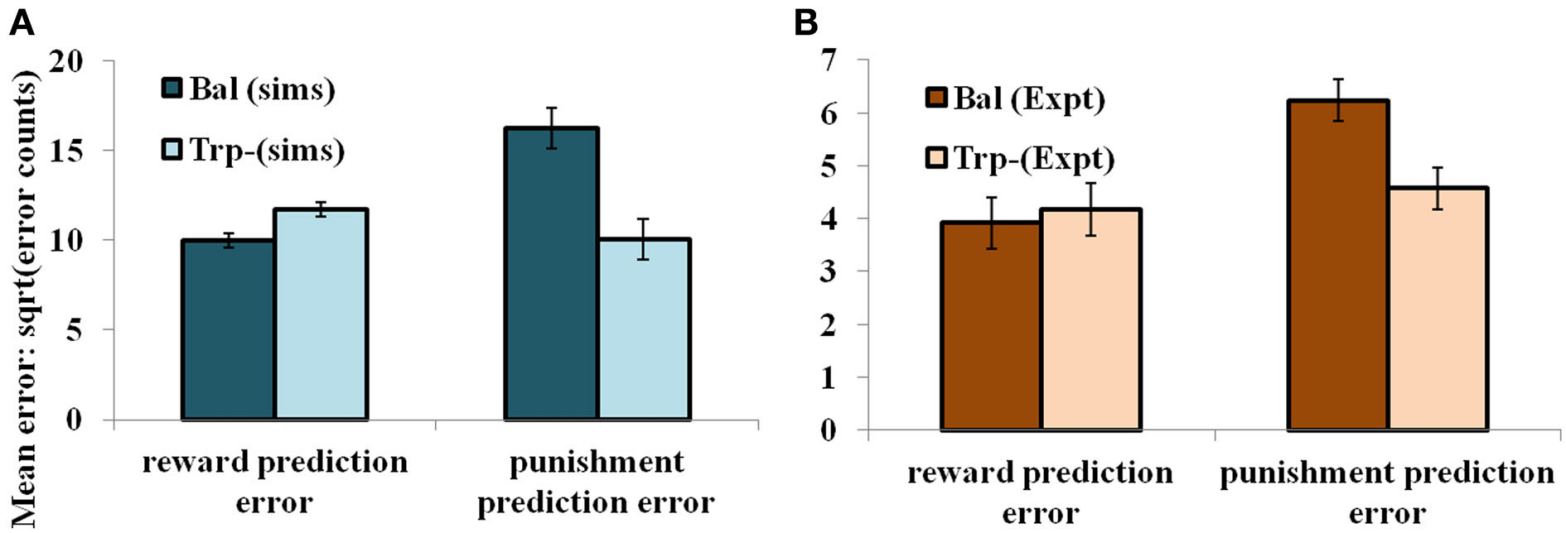
**The mean number of errors in non-switch trials (A) as a function of “α” and outcome trial type; “α = 0.5” (balanced) and “α = 0.3” (Tryptophan depletion).** Error bars represent standard errors of the difference as a function of “α” in simulation for size “*N*” = 100 (Sims). **(B)** Experimental error percentages adapted from Cools et al. (2008). Error bars represent standard errors as a function of drink in experiment (Expt). The results in **(B)** were reported after the exclusion of the trials from the acquisition stage of each block.

**Figure 5:**
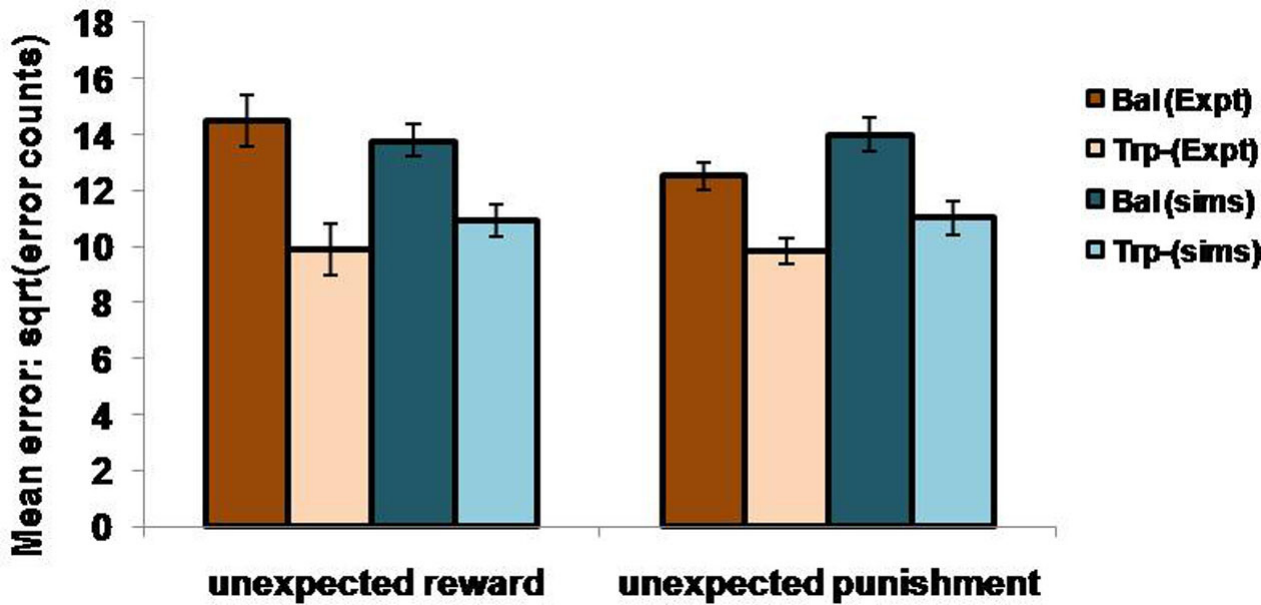
**The mean number of errors in non-switch trials as a function condition; Simulation (sims): “α = 0.5” (balanced) and “α = 0.3” (Tryptophan depletion).** Experimental (Expt) results adapted from Cools et al. (2008). Error bars represent standard errors either as a function of drink in experiment, or α in simulation for size “*N*” = 100.

In the non-reversal trials, all the errors with respect to the drink and the condition (viz., unexpected reward and unexpected punishment) are featured in the Figure 5. The errors with respect to the drink and the outcome (viz., reward and punishment prediction errors) in both conditions are shown in Figure 4. Our results (Figure 4: sims values) show that the reward prediction error in the simulations does not vary much from the balanced (optimized α = 0.5 representing control tryptophan) condition to the tryptophan depleted (represented by optimized α = 0.3) condition, but the punishment prediction error decreases thereby matching the experimental results [Figure 4: expt values adapted from Cools et al. (2008). The errors in unexpectedly rewarding and punitive trials are obtained to be the same in both the balanced and tryptophan depleted cases (Figure 5: sims values) again matching with the experiment [Figure 5: expt values adapted from Cools et al. (2008)]. Therefore, increased 5HT levels in balanced condition are seen promoting the inhibition of responses to punishing outcomes as proposed by Cools et al. (2008). Reducing 5HT via tryptophan depletion then removes this inhibition. We can see a similar result from (Figures 4, 5) depicting balanced (*α* = 0.5) and the tryptophan depleted (α = 0.3) conditions. *Sign*(*Q_t_*) term in Equation (3.3.2.5) plays a crucial role in this differential response to gains (rewards) and losses (punishments) (analysis of the results on removing the *Sign*(*Q_t_*) term is provided in Supplementary material). As the data is in the form of counts, the errors are reported as SQRT (error counts) (Cools et al., 2008) in Figures 4, 5.

## Discussion

### Main findings of the model

Reinforcement Learning framework has been used extensively to model the function of basal ganglia (Frank et al., 2007; Chakravarthy et al., 2010; Krishnan et al., 2011; Kalva et al., 2012). The starting point of our model was to understand the contributions of serotonin in BG function (Tanaka et al., 2009; Boureau and Dayan, 2011). We use the notion of risk, since serotonin is shown to be associated with risk sensitivity. Some instances are as follows: On presentation of the choices with risky and safe rewards, the reduction of central serotonin levels favor the selection of risky choices comparative to the baseline levels (Long et al., 2009). The non-linearity in risk-based decision making—risk aversivity in the case of the gains and risk seeking in the case of losses, is postulated to be affected by central serotonin levels (Murphy et al., 2009). Negative affective behavior such as depression, anxiety and other behavior such as impulsivity caused due to the reduction of the central serotonin levels, is argued to be a risky choice selection in a risk based decision making framework (Dayan and Huys, 2008). Based on the putative link between serotonin function and risk sensitivity, we have extended the classical RL approach of policy execution using the utility function (Equation 2.8) instead of value function. The utility function combines value function with risk function. We propose that the weightage (α) that combines value and risk in the utility function represents serotonin (5HT) functioning in BG. Using this formulation, we show that three different experimental paradigms instantiating diverse theories of serotonin function in the BG can be explained under a single framework.

The proposed model is applied to different experimental paradigms. The first is a bee foraging task in which bees choose between yellow and blue flowers based on the associated risk (Real, 1981). The proposal model is applied to this simple instance of risk based decision making, though the experiment does not particularly relate to DA and 5HT signaling. The risk sensitivity reported in the bee foraging experiment is predicted by our model (for α = 1) accurately.

Next we model experiments dealing with various functions of 5HT. One such experiment links 5HT levels to risky behavior. Experiments by Long et al. (2009); Murphy et al. (2009) discuss associating 5HT levels to non-linear risk sensitivity in gains and losses. In our study, we model a classic experiment by Long et al. (2009) describing the risk sensitivity in monkeys on depleting 5HT level. With our model, the effect of increased risk-seeking behavior in RTD condition is captured with parameter α = 1.658 and the baseline condition with α = 1.985. This result shows that our model's 5HT-correlate “*α*” can control risk sensitivity.

The third experiment is a reward prediction problem (Tanaka et al., 2009) associating 5HT to the time scale of prediction. Herein the subjects chose between a smaller short-term reward and a larger long-term reward. Our modeling results show that for a fixed γ, increasing α increases the probability of choosing the larger, long-term reward. Since higher α denotes higher 5HT level, the model corroborates the experimental result, suggesting that our model's 5HT-correlate “α” behaves similar to the time scale of reward prediction.

Finally the fourth experiment is to show the differential effect of 5HT on the sensitivity to reward and punishment prediction errors. Under conditions of balanced 5HT (α = 0.5), the model is less sensitive to punishment and commits more errors in predicting punishment; this trend is rectified in depleted 5HT (α = 0.3) condition. For numerical analysis of reward and punishment prediction error, the experiment by Cools et al. (2008) did not take the acquisition trials into consideration. However, these trials serve to learn the initial association between stimulus and response. They also act as a base for the forthcoming reversal and switch trials and are hence taken into analysis in our simulation. This differential effect shown by the model 5HT-correlate “α” toward punishment corroborates the experimental evidence linking 5HT to adverse behavior exhibited in psychological disorders like depression and anxiety (Cools et al., 2008, 2011; Boureau and Dayan, 2011).

Simulation results thus show that the proposed model of 5HT function in BG reconciles three diverse existing theories on the subject: (1) risk-based decision making, (2) time-scale of reward prediction and (3) punishment sensitivity. To our knowledge this is the first model that can reconcile the diverse roles of serotonin under a simple and single framework.

### Significance of sign(Q_t_)

The *sign(Q_t_)* term presented in the modified formulation of utility function (Equation 2.8) denotes the preference for risk in a given context of the experiment. At high mean reward values humans are found to be risk-averse, whereas at low mean reward values they are risk-seeking (Kahneman and Tversky, 1979). In neuroeconomic experiments, this risk preference is statistically determined, for example, by maximizing the log likelihood of the decisions (D'acremont et al., 2009). Though this method estimates the risk preference subjectively, it is derived from decisions made throughout the experiment. The use of *sign(Q_t_)* in our model takes into account the variation of the subjective risk preference, according to the expected cumulative reward outcomes observed *within* an experiment. The significance of this term in the formula of modified utility (Equation 2.8) can be seen from the Supplementary material. This Supplementary material presents the results of simulating the experiment by Cools et al. (2008) with an altered model having no *sign(Q_t_)* term in the utility function of Equation (2.8). The mean number of errors does not vary as a function of both trial type and condition, for different values of “α,” contrary to what happens in the experiment. Thus *sign(Q_t_)*term is essential for simulating the results of Cools et al. (2008). Such a behavior of nonlinear risk sensitivity has been shown to be modulated by 5HT in various experiments (Long et al., 2009; Murphy et al., 2009), which further strengthens our proposal of introducing the term *sign(Q_t_)* in Equation (2.8).

### 5HT-DA interaction in the “risk” component of decision making

The risk part of the utility function (Equation 2.8) has three components: α, *sign(Q_t_)*and √*h_t_*. While “α” represents 5HT, the remaining two components are dependent on “δ ” or DA. Thus the proposed model of risk computation postulates a complex interaction between DA and 5HT. In neurobiology, complex interactions are indeed seen to exist between DA and 5HT (Di Matteo et al., 2008a,b) at the cellular level that are not detailed in this present abstract model. The 5HT afferents from dorsal raphe nucleus differentially modulate the DA neurons in SNc and ventral tegmental area (VTA) (Gervais and Rouillard, 2000). The 5HT projections act via specific receptor subtypes in the DA neurons. Action of 5HT 1A, 5HT 1B, 5HT 2A, 5HT 3, 5HT 4 agonists facilitate dopaminergic release, whereas 5HT 2C agonists inhibit the same. Selective serotonin reuptake inhibitors are known to reduce the spontaneous activity of DA neurons in VTA (Di Mascio et al., 1998; Alex and Pehek, 2007; Di Giovanni et al., 2008). The 5HT neurons in Dorsal Raphe nucleus also receive dense DA innervations from midbrain DA neurons (Ferre et al., 1994) and express D2R (Suzuki et al., 1998).

### Contributions from existing models

The previous models on 5HT seem to focus on individual functions of 5HT in isolation without reconciling them in a single framework. Most of them consider 5HT as a neuromodulator mediating aversive outcomes (Daw et al., 2002; Boureau and Dayan, 2011; Cools et al., 2011). Some describe 5HT as a controller of time-scale in prediction of rewards (Tanaka et al., 2007), and as a modulator that associates the aversive outcomes to past actions (Tanaka et al., 2009). Psychological disorders associated with lowered 5HT levels, such as impulsivity and negative moods, have also been studied by the existing models on 5HT. They infer impulsivity to be the result of increased short term reward prediction (Tanaka et al., 2007), and negative moods to increased punishment sensitivity, respectively (Cools et al., 2011; Robinson et al., 2012). Such observation may then be captured in our model by assessing the risk involved in the task and by controlling the “α” (5HT) parameter.

### Study predictions and future work

Our proposed unified model is an abstract mathematical model, aimed at explaining a range of behavioral effects of 5HT. It is only a preliminary model that uses a modified RL framework and explains the role of 5HT and DA in the BG. It focuses mainly on risk computation and the role of nigrostriatal DA signal in shaping the learning of risk and value in BG. Ideally, a convincing model of utility computation in BG should go beyond the 5HT-DA interaction in the abstract representation of the value and the risk quantities and demonstrate how the utility computation would be carried out by the neurobiological correlates in BG.

In classical Actor-Critic approaches to modeling BG function, value computation is thought to occur in striatum (Joel et al., 2002). There is evidence from functional imaging that supports this theory (O'doherty et al., 2006). There is strong evidence for the existence of DA-modulated plasticity in corticostriatal connections, an effect that is necessary to account for value computation in the medium spiny neurons (MSNs) of striatum (see review by Kötter and Wickens, 1998). The idea that MSNs are probably cellular substrates for value computation has found its place in recent modeling literature (Morita et al., 2012).

Starting from the fact that the effect of DA on the D1-expressing MSNs of the striatum is to increase the firing rate (by having an increasing gain as a function of δ), it has been shown in a computational model of BG that these D1-expressing MSNs are capable of computing value (Krishnan et al., 2011). Just as D1R-expressing MSNs are thought to be cellular substrates for value computation in the striatum (Kötter and Wickens, 1998; O'doherty et al., 2006; Krishnan et al., 2011; Morita et al., 2012), we propose that D1D2-coexpressing MSNs can be the cellular correlates for risk computation. We have already developed a network model of BG in which risk is computed by D1D2-coexpressing neurons in the striatum, while value is computed by D1-expressing medium spiny neuron (unpublished). Just are neurons that compute value function (Equations 2.3–2.4) require monotonically increasing gain as a function of δ in the MSNs, risk function (Equations 2.6–2.7) would require a “U-shaped” gain function as a function of δ. It is plausible that these risk-type of gain functions would then probably be exhibited by neurons that coexpress both the D1-like gain function that increases as with δ, *and* D2-like gain function that decreases with δ (Servan-Schreiber et al., 1990; Moyer et al., 2007; Thurley et al., 2008; Humphries et al., 2009). Interestingly about 59% neurons in Globus Pallidus and 20–30% in ventral striatum coexpress D1R and D2R (Perreault et al., 2010). Even among the MSNs of the striatum, the proportion of D1R-D2R co-expressing neurons are greater in ventral striatal MSNs (17% in shell) compared to 5% in dorsal striatum (Surmeier et al., 1996; Bertran-Gonzalez et al., 2008). Some studies also point out that around 70% of the MSNs in striatum coexpress the D1 and the D2 type receptors (Surmeier et al., 1996). The ventral striatum also mediates risk sensitivity in action selection (Stopper and Floresco, 2011), the latencies of response, and the sensitivity to the magnitude of the rewards (Acheson et al., 2006; Floresco et al., 2006). This encourages us to predict a link between the risk-based functioning of the ventral striatum and the significant presence of the co-expressing D1R-D2R neurons here. We would also like to explore the plausibility of the functioning of D1R-D2R co-expressing neurons to the computation of the risk function and the selective modulation of serotonin on these risk computing neurons in future. We predict therefore that selective loss of these co-expressing neurons would make the subject less sensitive to the risk component of the environment.

The role of serotonin in reward and punishment sensitivity of PD subjects could also be analyzed using our proposed modeling approach. In experiments where reward/punishment sensitivity of PD subjects was studied, PD patients ON DA medication showed an increased reward sensitivity compared to PD OFF subjects who showed increased punishment sensitivity (Frank et al., 2007; Bodi et al., 2009). Our proposed model, in which serotonin controls the weightage of risk, is expected to account for the aforementioned experimental results. Preliminary work on application of the proposed model to the study of (Bodi et al., 2009) gave encouraging results (unpublished).

In connection with the neurobiological correlate of the -*sign(Q_t_)* term, the aforementioned discussion suggests a general, complex interaction between DA and 5HT signals. But as a specific circuit that can form the basis for the -*sign(Q_t_)* term in Equation 2.8, we invoke the circuitry that links habenula with striatum. Habenula is a structure that is thought to be involved in brain's responses to reward, pain and anxiety (Lecourtier and Kelly, 2007; Hikosaka, 2010). It gained importance for its interactions with the DA and 5HT systems (Lecourtier and Kelly, 2007; Hikosaka, 2010). It is a small structure located near the posterior-dorsal-medial end of thalamus. It is divided into medial habenula (MHb) and lateral habenula (LHb). Striatum (in particular D1R containing striosome) and LHb are thought to form a negative feedback loop [LHb→Rostromedial Tegmental Nucleus (RMTg)→VTA/SNc→Striatum→Globus Pallidus→LHb], not via direct connections but via intermediaries (Lecourtier and Kelly, 2007; Hikosaka, 2010). Activation of LHb neurons inhibits the DA cells of VTA and SNc. This DA is also known to have a special action on MSNs as follows. Activation of D1 receptors is known to enhance (suppress) the activation of MSNs if the prior membrane state is depolarized (polarized) (Flores-Hernandez et al., 2002). However, we do not know if the action of DA on the hypothesized risk computing D1–D2 co-expressing neurons is one of the stabilizers of the pre-existing state. Based on the data reviewed above, we plan to develop a model in which D1-expressing MSNs whose activity represents value, act on D1R-D2R co-expressing MSNs via habenula, by an interaction term that can be roughly described by—*sign(Q_t)_*.

Finally, a theory of 5HT and DA in the BG must go beyond the striatum since 5HT innervations in the BG are not confined to striatum, but include GPe, SNc, and PPN (Wallman et al., 2011). We plan to elucidate the role of 5HT and DA in these other nuclei of the BG through a more complete network model in our future. The suggested roles of DA in the BG include, (1) plasticity of corticostriatal connections, (2) switching between DP and IP by striatal DA, and (3) modulating the exploratory drive arising from the STN-GPe system (Chakravarthy et al., 2010; Kalva et al., 2012). Analogously, a comprehensive theory of 5HT and DA in the BG is planned to be developed. The theory might shed light on the role of 5HT in some of the key functions of the BG viz., action selection/decision making.

## Conflict of interest statement

The authors declare that the research was conducted in the absence of any commercial or financial relationships that could be construed as a potential conflict of interest.
