# Supplementary material for: An extended reinforcement learning model of basal ganglia to understand the contributions of serotonin and dopamine in risk-based decision making, reward prediction, and punishment learning
Source: Front Comput Neurosci. 2014 Apr 16;8:47. doi: 10.3389/fncom.2014.00047 (PMC3997037; doi:10.3389/fncom.2014.00047)
Supplement: Supplementary file 1 [file DataSheet1.DOCX]

**SUPPLEMENTARY MATERIALS:**

**Supplementary Material A:**

The Genetic Algorithm([Goldberg, 1989](#_ENREF_2)) option set for optimization is given in the following table. Optimization toolbox 6.0, Matlab R2011a, The Mathworks Inc. is used.

Option set for the GA tool

| **Option** | **Value** | | | |
| --- | --- | --- | --- | --- |
| Population Size | 20 | | | |
| Crossover fraction | 0.8 | | | |
| Elite count | 4 | | | |
| Generation time | 1000 | | | |
| Function tolerance | 1 e-6 | | | |
| Bounds |  | ***α*** | ***r_b_*** | ***β*** |
|  | Upper | 5 | 300 | 100 |
|  | Lower | -5 | 0 | 0 |
| Cost function | (Expt measure - Sims measure)^2^ | | | |

**Supplementary Material B**:

Time scale of reward prediction and serotonin:


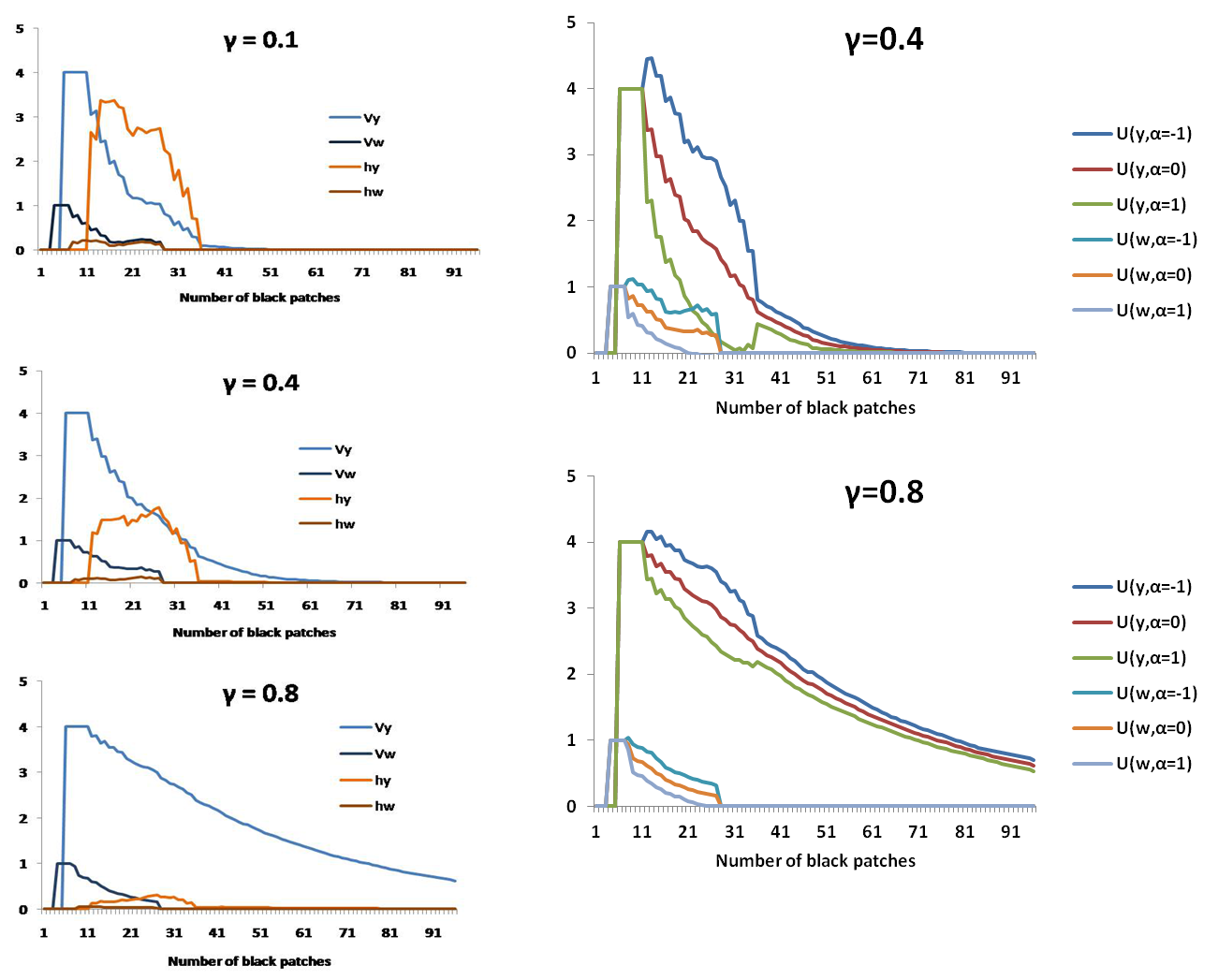


The simulated value (*Q*) and the risk (*h*) functions across the state space for different values of *γ*. 'w'denotes the white panel and 'y' denotes the yellow panel. (a) *γ* = 0.1; (b) *γ* = 0.4; (c) *γ* = 0.8; and the simulated utility (*U*) values of α = [-1, 0 ,1] for (d) *γ* = 0.4; (e) *γ* = 0.8;

**Supplementary Material C:**


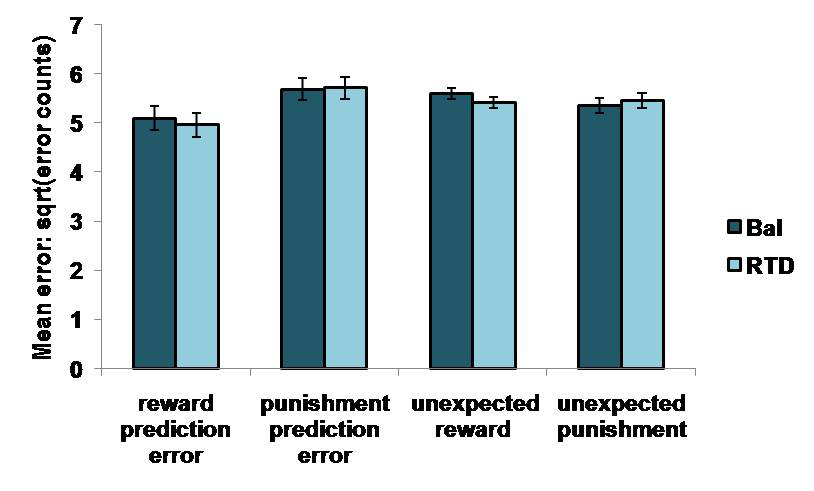


The mean number of errors in non-switch trials as a function of '*α*' and outcome trial type, condition; '*α* = 0.5' (balanced)and '*α* = 0.3' (Tryptophan depletion). Error bars represent standard errors of the difference as a function of α with N= 100. The Figure shows the result of simulating the experiment by ([Cools et al., 2008](#_ENREF_1)) with an altered model having no *sign(Q_t_)* term in the utility function of eqn. 2.8. There was no difference seen in the mean number of errors both as a function of trial type and condition, on varying the values of *α*.

**REFERENCES**

Cools, R., Robinson, O.J., and Sahakian, B. (2008). Acute tryptophan depletion in healthy volunteers enhances punishment prediction but does not affect reward prediction. *Neuropsychopharmacology* 33**,** 2291-2299.

Goldberg, D.E. (1989). *Genetic Algorithms in Search Optimization and Machine Learning.* Addison-Wesley Longman Publishing Co.,.
